# The Sustainable Personalised Interventions for Cognition, Care, and Engagement (SPICE) program: Pre and post outcomes of a multicomponent intervention for people with dementia and care partners

**DOI:** 10.1002/alz70858_107464

**Published:** 2025-12-26

**Authors:** Nathan M D'Cunha, Georgina Chelberg, Rachael Mitterfellner, Lara Wiseman, Kasia Bail, Stephen Isbel, Stephanie Mulhall, Helen Holloway, Sam Kosari, Louise Barrett, Michelle Bennett, Diane Gibson

**Affiliations:** ^1^ University of Canberra, Bruce, ACT, Australia; ^2^ Canberra Health Services, Bruce, ACT, Australia

## Abstract

**Background:**

Access to comprehensive post‐diagnostic support is crucial to promote quality of life (QoL), and cognitive and physical function following dementia diagnosis. The Sustainable Personalised Interventions for Cognition, Care, and Engagement (SPICE) program aims to meet the need for community‐based care and rehabilitation by delivering a multidisciplinary, multicomponent approach for people with dementia and care partners in an outpatient hospital setting.

**Method:**

This study evaluates the 12‐week SPICE program using a pragmatic waiting‐list design. The SPICE program includes both group (Cognitive Stimulation Therapy, care partner education, and physical activity) and dyadic (dietary advice, and the Care Of People with dementia in their Environments [COPE]) interventions. Here we report findings of key outcome measures, Dementia QoL (DEMQOL) and care partner DEMQOL, Addenbrooke's Cognitive Examination (ACE‐III) and Neuropsychiatric Inventory‐Questionnaire (NPI‐Q) assessed pre‐intervention and post‐intervention. Follow‐up data collection will be completed in March 2025.

**Result:**

Ninety‐one people with dementia (mean age 78.2 ± 6.5, range 57–94; 39.2% female) and 91 care partners (mean age 71.6 ± 10.8, range 37–91 years; 60.8% female) completed the intervention across 14 groups, each containing 6‐7 dyads. There were significant differences in QoL for people with dementia with DEMQOL scores increasing by a mean of 4.4 points (95% CI [2.36, 6.44]), DEMQOL‐Proxy by 5.91 (95% CI [3.51, 8.31]), ACE‐III mean scores increasing by 1.61 (95% CI [.35, 2.87]), and NPI‐Q scores decreasing by a mean of 1.76 points (95% CI [.80, 2.72]). Significant mean differences were also observed for physical outcomes for people with dementia and care partners, including on the Alternate Step Test, Timed Up and Go, and 10‐Metre Walk Test.

**Conclusion:**

The SPICE program demonstrates that post‐diagnostic support is feasible and associated with improved outcomes for people with dementia and care partners. Broader implementation and adaptations to support sustainability are warranted.

